# Methodology for eliciting consensus on high-level concepts in rare connective tissue and musculoskeletal diseases (ReCONNET-ELICIT)

**DOI:** 10.1186/s13023-026-04502-3

**Published:** 2026-07-15

**Authors:** Laurent Arnaud, Guillermo Pons-Estel, Mariele Gatto, Chiara Tani, Hervé Devilliers, Rosaria Talarico, Giuseppe Turchetti, Marta Mosca

**Affiliations:** 1 Department of Rheumatology, National Reference Center for Rare Autoimmune Diseases (RESO), Hôpitaux Universitaires de Strasbourg, Strasbourg, France; 2 Regional Center for Autoimmune and Rheumatic Diseases (CREAR), Rosario, Argentina; 3 Academic Rheumatology Centre, Department of Clinical and Biological Sciences, University of Turin, AO Mauriziano, Turin, Italy; 4 Department of Clinical and Experimental Medicine, University of Pisa, Pisa, Italy; 5 Internal Medicine and Systemic Diseases Unit, University Hospital Centre Dijon, Dijon, France; 6 Rheumatology Unit, Azienda Ospedaliero Universitaria Pisana, University of Pisa, Pisa, Italy; 7 Institute of Management, Scuola Superiore Sant’Anna, 56127 Pisa, Italy

## Abstract

The ReCONNET-ELICIT methodology represents an innovative and systematic approach to gather expert consensus on complex, high-level concepts related to rare connective tissue and musculoskeletal diseases (rCTDs). Developed within the framework of European Reference Networks (ERNs) ReCONNET, this methodology aims to overcome the challenges associated with defining complex features that lack consensus in rCTDs, such as composite definitions for severe or refractory disease. The core premise of RECONNET-ELICIT is that individual expertise, including that of experts, is inherently limited, but that aggregating insights from a sufficiently large and diverse group can achieve extensive and relevant facet elicitation. The RECONNET-ELICIT process begins with assembling a diverse panel from various geographical backgrounds. This inclusive approach ensures that multiple perspectives are considered. The next step involves crowdsourcing responses through an online survey, where participants suggest as many facets of the high-level concept as possible. These proposals are then systematically analyzed and recoded into broader categories through a consensus-driven binning process. Following this, the panel reviews the consolidated list of facets and votes on their final inclusion, typically utilizing rating scales to minimize bias. This approach is particularly valuable in rCTD contexts where limited data are available. Overall, ReCONNET-ELICIT aim at enhancing the clarity and shared understanding of complex disease concepts by integrating expert insights within an asynchronous, structured, participatory framework while avoiding dominance bias. Its flexibility and focus on collective agreement make it especially useful for advancing knowledge and improving management strategies for rare CTDs.

## Key Message

**What is already known on this topic**

Rare connective tissue and musculoskeletal diseases (rCTDs) often lack standardized criteria and consensus definitions, especially for complex high-levels concepts such as severe, refractory disease or rare manifestations.

**What this study adds**

The ReCONNET-ELICIT methodology offers an innovative, systematic approach to gather expert participation and consensus on complex, high-level concepts in rCTDs, where robust evidence is typically lacking, leveraging diverse and comprehensive perspectives, from an expert panel.

**How this study might affect research, practice, or policy**

By systematically capturing facets of high-level complex concepts through expert consensus, the ReCONNET-ELICIT methodology can improve clinical guidelines, guide future research efforts and inform policy development, ultimately enhancing rCTD patient care.

## Introduction

European Reference Networks (ERNs) are virtual collaborations supported by the European Union, connecting healthcare professionals (HCPs) across Europe to address rare and complex diseases requiring highly specialized expertise [1]. The ERN ReCONNET, dedicated to Connective Tissue and Musculoskeletal Diseases, comprises 63 healthcare providers from 23 EU Member States and includes 15 patient representatives [2]. Through initiatives such as webinars, expert exchanges, the development of patient-friendly summaries of clinical guidelines [3], early diagnosis red flags, and innovative methodologies [4], ERNs aim to enhance understanding and management of rare connective tissue diseases (rCTDs).

These rCTDs encompass a broad spectrum of diseases, characterized by distinct manifestations, pathophysiological features, and clinical complexities. While classification criteria are generally established based on hallmark disease features, considerable variability exists in how disease activity, organ damage, and patient-reported outcomes are assessed [5]. Moreover, certain complex disease features, such as severe or refractory disease states, rare manifestations, typically lack standardized definitions and are difficult to define according to available data or existing literature [6].

Here, we introduce a novel methodology, termed ReCONNET-ELICIT, designed to systematically identify and define the various “facets” of high-level, complex disease concepts using expert insight. The term “facets” should be understood as representing the various dimensions, domains, or aspects of the high-level concept. It was chosen to avoid any potential confusion with the more restrictive concept of “organ-domains” in SLE. A “facet” is not, in itself, an observable indicator or measurement variable, a decision criterion or threshold, or a fully operationalized definition. Rather, facets are intended to operate at an intermediate, conceptual level to organize the construct into non-overlapping but complementary dimensions, each of which can subsequently be informed by empirical indicators or expert judgment.

This methodology operates on the premise that individual expertise, including that of experts, is inherently limited, but that aggregating insights from a sufficiently large and diverse group can achieve extensive facet elicitation. By aggregating expert feedback, this approach aims to delineate relevant features of complex concepts and reach consensus on their significance. Importantly, the outputs of the method reflect the scope of perspectives captured within the participating expert group, rather than an exhaustively complete representation of the conceptual domain. In this respect, and given the diversity of panel composition, elicited formulations may vary in their level of abstraction, with some participants expressing conceptual dimensions and others formulating closely related content in terms of indicators, thresholds, or operational criteria. No formal stopping rule or saturation criterion is applied in the current methodology, and the extent of coverage depends on factors such as panel size, diversity, and iteration. ReCONNET-ELICIT has previously been applied for defining rare lupus manifestations [6], severe disease states, and potential patient vulnerabilities along the care pathway, demonstrating its utility in advancing the understanding of complex disease phenotypes and optimal treatment strategies in rCTDs.

## General methodology

The general methodology for ReCONNET-ELICIT was proposed and drafted by one of the authors (L.A.) and enhanced through discussions with multiple stakeholders, including a methodologist (H.D.). The primary objective of this methodology is to produce a structured, transparent, and operationalized consensus on how a given panel conceptualizes and prioritizes a target concept. Unlike conventional literature reviews, the ReCONNET-ELICIT methodology incorporates a first step designed to identify the various facets of the high-level concept to be defined. This step emphasizes the importance of integrating expert opinion and *crowdsourcing* techniques to enhance the applicability of the high-level concept across diverse contexts. Here, we define “high-level concepts” as complex, composite, typically multidimensional constructs, involving heterogeneous clinical or contextual components that are not directly measurable, and often lack standardized or universally accepted definitions. The method is particularly suited to high-level concepts that can be decomposed into relatively discrete and operationalizable facets, allowing these components to be systematically elicited, recoded, and prioritized. Of note, the outputs of ReCONNET-ELICIT are panel-dependent and context-sensitive, reflecting the collective expertise and perspectives of the participating experts.

**Fig. 1:**
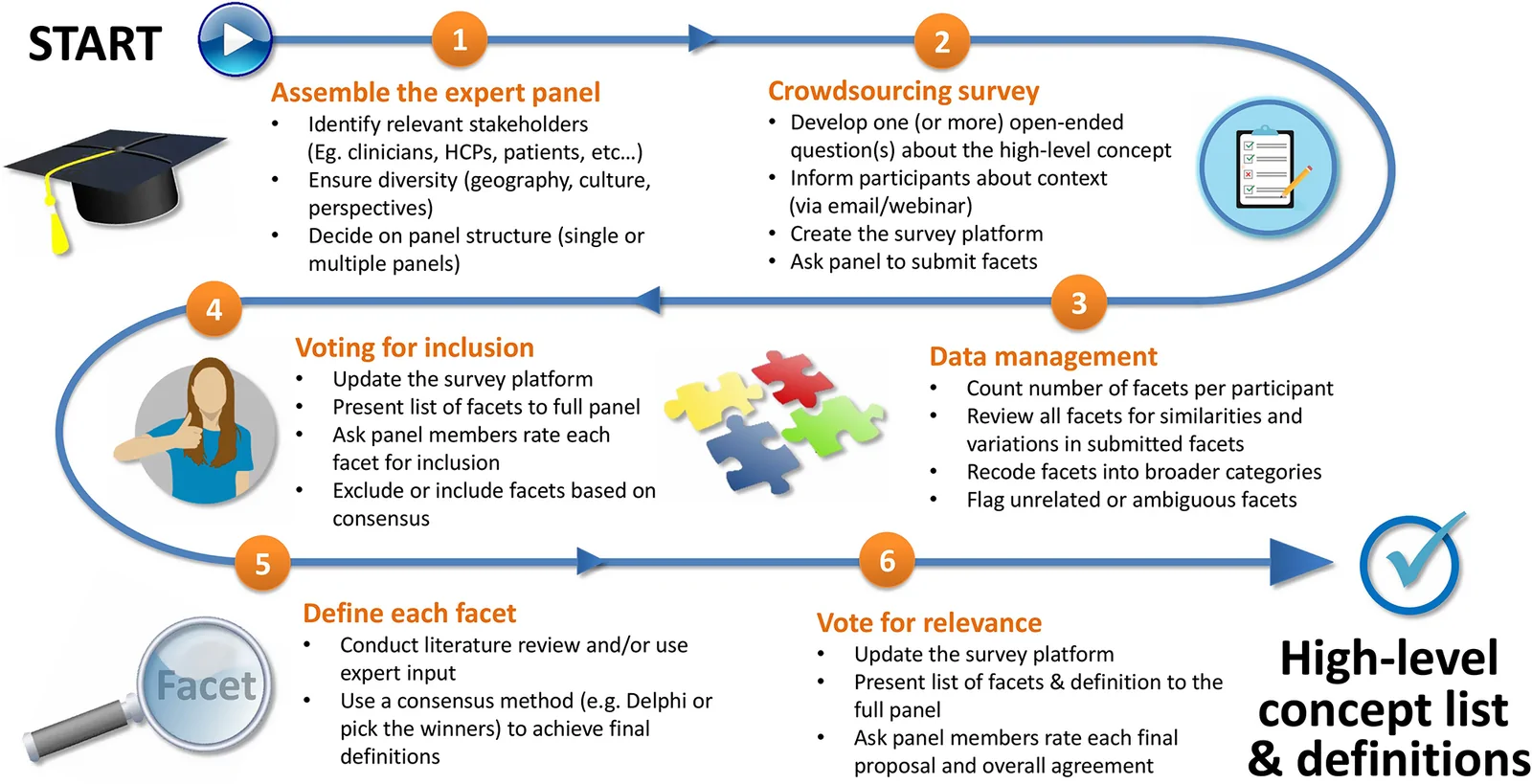
Overview of the ReCONNET-ELICIT methodology for eliciting collective feedback on high-level concepts

The ReCONNET-ELICIT process involves several steps (Fig. 1):

**The first step** of the ReCONNET-ELICIT methodology involves assembling an expert panel tailored to the research question. Depending on the study objectives, this panel may comprise clinicians, other healthcare professionals, experts in healthcare organization and health economics, and additional stakeholders such as representatives from pharmaceutical companies, payers, patients, or patient advocacy groups. Based on the primary aims of the study, participants can be grouped into a single panel or divided into multiple panels, particularly when comparing perspectives is relevant, such as between clinicians and patients. To thoroughly capture the various facets of the question, it is advisable to include a sufficiently diverse participant panel, typically exceeding 30 individuals, from different geographical regions and cultural backgrounds. This diversity ensures that multiple perspectives related to access to care, cultural norms and, when appropriate religious considerations, are represented. Of note, the outputs of ReCONNET-ELICIT are panel-dependent and context-sensitive, reflecting the collective expertise and perspectives of the participating experts. While the method is *primarily* oriented toward producing a shared structured framework, different stakeholder groups such as clinicians, other health professionals, and patients may bring not only complementary insights but also partially incompatible priorities, languages, and criteria of relevance. These differences can result in significant disagreement that we believe is informative rather than obstructive, revealing ambiguities inherent in the high-level concept under study. Reporting such divergences can provide valuable insights for subsequent research or clinical guideline development.

**The second step**, involves surveying the participant panel to gather a comprehensive set of potential high-level concept facets. We recommend avoiding the use of the term “domain” instead of “facet” as the former may be misinterpreted as organ-specific domains within the context of rCTDs. A facet is a distinct conceptual dimension of the target construct that captures a specific aspect along which the construct can be meaningfully described or differentiated. Importantly, a facet is necessary to structure the concept but is not, on its own, sufficient to define the construct, and should not be understood as an observable indicator or measurement variable, a decision criterion or threshold or a fully operationalized definition. Facets organize the construct into non-overlapping but complementary dimensions, each of which can subsequently be informed by empirical indicators or expert judgment.

**Fig. 2:**
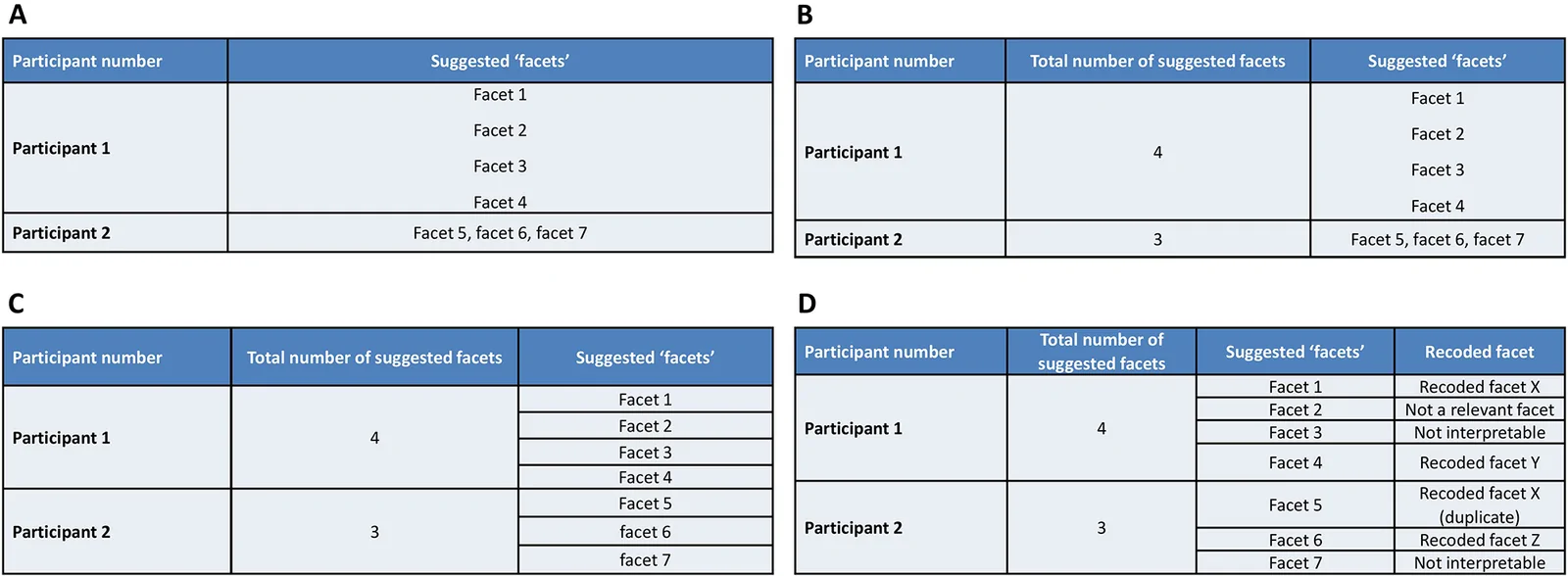
Quantification and recoding of the facets associated with the high-level concept, provided by each participant. Panel A showing raw data formatting, as entered by participants; note that participant 1 followed the instruction to split submitted facets across multiples lines, while participant 2 submitted them as a single line. Panel B showing the number of facets submitted by each participant. Panel C showing facet splitting across different lines. Panel D showing recoded facets; note that facet 2 is not relevant to high-level concept, that facet 5 was similar to facet 1, and that facet 7 was not interpretable

This step is typically conducted via an online platform, where participants are asked one (or less commonly several) open-ended questions designed to elicit as many facets of the high-level concept as possible. To facilitate meaningful participation, it is recommended to inform participants about the context and the primary question prior to the survey. This can be accomplished through an introductory email, a preliminary webinar, or a preparatory meeting. Encouraging participants to reflect on the concept beforehand can enhance the quality and depth of their responses. Participants can be advised to compile their answers externally over several days, for example, and to submit them later into the digital platform. Of note, while no formal literature review is embedded as a mandatory initial step, prior knowledge is not absent in practice, as participating experts typically bring substantial familiarity with the field. In addition, the incorporation of a preliminary systematic or scoping review may be considered as an optional, context-dependent step to complement expert elicitation and to reduce the risk of omission of previously described elements. To facilitate data management (cf. below), it is advisable to instruct participants to report each proposed facet on a separate line within the survey (see Panel A of Fig. 2). This approach simplifies subsequent data processing and analysis. To optimize participation rates, it is recommended to allocate sufficient time for survey completion and to send periodic reminder emails to non-responders.

**The third step** involves quantifying the number of facets, associated with the high-level concept, provided by each participant. The recoding step represents a critical phase of the ReCONNET-ELICIT methodology, in which heterogeneous expert contributions are transformed into a structured set of votable facets. This process involves including decisions regarding semantic equivalence and conceptual proximity. This process begins with counting the number of submitted facets per participant and adding this information to a new column in the data table (see Panel B of Fig. 2). Subsequently, each participant data is duplicated across as many rows as of suggested facets, and the corresponding facet descriptions are then split across these multiple rows (see Panel C of Fig. 2). Next, the facets are consolidated into broader categories through windowing and binning, reducing the data to a manageable number of categories. To do this effectively, the assessor should first review all responses to grasp the overall sense and distribution of proposals and identify any important variations in the data. The assessor then adds another column to the data table (see Panel D of Fig. 2) and recodes each facet into a limited set of categories. This consists in grouping participant formulations under common facet labels when they referred to the same underlying aspect of the construct. These formulations could differ in their logical and epistemic status, ranging from conceptual expressions to qualified states, empirical indicators, thresholds, or composite operational criteria. Accordingly, this step entails an interpretive transformation, whereby elements of differing logical status are aligned under a shared conceptual referent. In this sense, the recoding does not merely consolidate semantically proximate statements, but also bridges distinct levels of formulation present in the source material. This approach allows the analytical framework to retain conceptual coherence while remaining grounded in the diversity of expert expressions elicited during the process. Special attention should be paid to identifying duplicate entries or related concepts to ensure consistent grouping. During this process, any facets that are unrelated to the primary survey question or are ambiguous or difficult to interpret should be clearly flagged as such. Distinct formulations are retained rather than merged if they reflect substantively different conceptual perspectives. Whenever possible, this coding phase should be performed independently by two assessors. The assessments are then compared, and discrepancies are resolved through discussion to reach consensus. In cases of persistent disagreement, a third assessor can be involved to facilitate resolution. To optimize efficiency in case of multiple assessors, the first one may initially review a subset of 50 to 100 responses to gain insight into common responses and develop a preliminary list of recoding categories which will help streamline the subsequent recoding process.

The rationale for adopting this formatting approach is to facilitate straightforward calculation of key metrics, including the total number of participants, the total number of facets submitted by the panel (encompassing raw, non-informative, duplicate, and informative entries), as well as the median number of facets proposed per participant. This structure allows for efficient data analysis across different participant panels, easily supporting comparison between relevant groups of interests, if appropriate.

Table 1 illustrates the recoding step and highlights that participant contributions may take different forms, including conceptual formulations, indicators, and operational criteria. The table should therefore be read as an illustration of how heterogeneous expressions are interpreted and grouped under shared facet labels, rather than as a set of elements all possessing the status of conceptual dimensions. Particular attention is therefore paid to preserving meaning during aggregation, and distinct formulations are retained when they reflect substantively different conceptual viewpoints. Of note, expert participants tend to use a relatively standardized technical lexicon. This may facilitate the production of closely aligned formulations for similar facets, thereby supporting the recoding process, although it may also reflect a prior degree of epistemic convergence within the expert community. Accordingly, such lexical standardization should not in itself be considered a safeguard against bias during recoding, nor against the potential reduction of minority or non-standard formulations. While procedures such as independent coding, comparison, and consensus-based reconciliation are implemented, this step remains subject to potential biases, including risks of semantic reduction. Further, it is worth noting that outputs are contingent on the interpretive framework applied by the assessors.

**Table 1:** Illustration of the recoding process in the identification of facets for the high-level concept of “severe” systemic lupus. The table presents examples of participant formulations in their original wording (step 1) and the corresponding extracted facets. These formulations are heterogeneous in nature, ranging from conceptual expressions (e.g., “level of disease activity”) to qualified states (e.g., “high disease activity”), empirical indicators (e.g., disease activity scores), and operational criteria (e.g., “SLEDAI > 10” or composite definitions). Their grouping under common facet labels reflects the interpretive recoding step, whereby formulations referring to a shared underlying aspect of the construct are aligned under a common conceptual referent

Examples of participant formulations (step 1)	Extracted facet
“high disease activity”	DISEASE ACTIVITY
“severe disease activity”	DISEASE ACTIVITY
“elevated and reversible inflammatory activity in organs”	DISEASE ACTIVITY
“SLEDAI > 10”	DISEASE ACTIVITY
“level of disease activity”	DISEASE ACTIVITY
“High Disease Activity Scores”	DISEASE ACTIVITY
“SLEDAI2K score of > 12 and/or at least one BILAG A score”	DISEASE ACTIVITY
“Damage potential”	DAMAGE / RISK OF DAMAGE
“existing DAMAGE”	DAMAGE / RISK OF DAMAGE
“eliciting permanent damage”	DAMAGE / RISK OF DAMAGE
“SLE with damage”	DAMAGE / RISK OF DAMAGE
“may result in organ damage”	DAMAGE / RISK OF DAMAGE
“REFRACTORY to treatment”	REFRACTORY DISEASE
“SLE refractory to treatment”	REFRACTORY DISEASE
“lack of response to other immunosuppressants”	REFRACTORY DISEASE
“requirement to change many treatments (> 2)”	REFRACTORY DISEASE
“medication resistance”	REFRACTORY DISEASE
“Refractory Disease (resistance to standard therapy)”	REFRACTORY DISEASE

**The fourth step** relies on the recognition that some of the facets identified in earlier phases, particularly if reported by only a minority of participants, may not be fully relevant to the study context. Conversely, it is also possible that the group consider a concept submitted by a single participant highly relevant. During this phase, the final list of potential facets from the previous steps is presented to the full panel. If necessary, the panel may be expanded to include additional experts. We recommend presenting the list of potential facets without disclosing the number of participants who reported each of these facets in the earlier phases, to minimize bias. Participants are then asked to rate each potential facet using a provided scale, indicating whether they wish to include it in the final set. Although there is currently no consensus on the optimal scale format, previous studies have commonly employed cutoffs such as ratings of ≥ 7 or 8 on a 0–10 scale, or scores ≥ 7 on a 1–9 scale, to determine inclusion. Importantly, this step should not be interpreted as establishing conceptual validity in a strong epistemic sense. Rather, it reflects structured group convergence under defined conditions, indicating which facets are collectively retained by a given expert panel. As such, voting captures agreement, not necessity, sufficiency, or universal validity of the underlying concept.

**Fig. 3:**
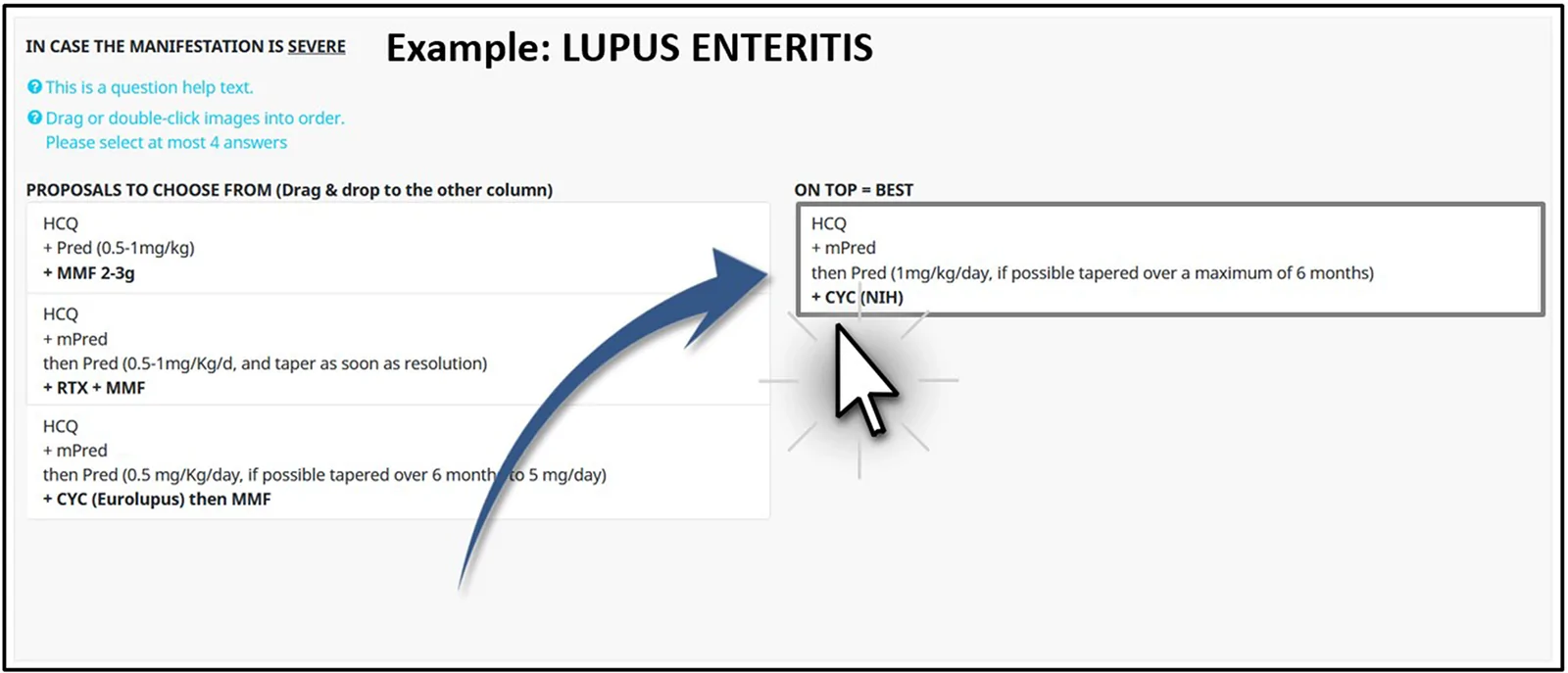
Example of “pick-the-winners” using the drag-and-drop feature

**The fifth step** is essential for defining each facet by consensus. While this process typically involves a systematic or targeted literature review followed by a consensus exercise (e.g., using the Delphi methodology), it is important to note that, in the specific context of the ERN ReCONNET-SLICC-SLEuro Expert consensus on rare lupus manifestations [6], where high-level evidence was inexistent, the consensus task force adopted an alternative approach. The primary objective at this stage was to identify potential therapeutic strategies for each rare lupus manifestation. To facilitate this, we developed a digital platform that allowed expert clinicians with extensive experience managing these rare manifestations to submit proposals for therapeutic strategies. Submissions were refined through rewording for clarity, as well as winnowing and binning. The curated list was then subjected to a voting process using a “pick-the-winners” approach among the full panel. For this, participants used an interactive drag-and-drop feature (Fig. 3) to select and rank the most relevant strategies for each rare manifestation, considering disease severity, among the proposals. The strategies that received the highest consensus votes were extracted as the most appropriate options for each specific rare disease manifestation and severity level. This concept can be extended to diverse context, such as selecting the most-fit definition for each domain of the high-level context. In another example (LUPUS-ALERT study), the ReCONNET-ELICIT methodology was used to assemble and define a list of relevant socio-determinants of health, which may impact SLE care pathways.

In the ERN ReCONNET-SLICC-SLEuro Expert consensus on rare lupus manifestations [6], a total of four potential strategies were suggested for the treatment of severe lupus enteritis, and the participants selected the 4th option offered as the most relevant one.

**Finally**,** as the sixth step**, it is recommended to request the panel to vote on whether the finalized list of high-level concepts and their corresponding definitions align with the primary objectives of the study.

The ReCONNET-ELICIT methodology is different from traditional consensus methods such as the nominal group technique and the Delphi method [7] through its unique process of crowdsourcing facets of a high-level concept from an expert panel, followed by facet selection by the group (Table 2).

**Table 2:** Core differences between the Delphi method, the nominal group technique, and the ReCONNET-ELICIT methodology

Consensus method & process	Delphi method	Nominal Group Technique (NGT)	ReCONNET-ELICIT
Core principle	Achieve consensus on pre-defined items through iterative rounds	Prioritize and rank items through structured group discussion	Elicit and structure high-level concept facets, then achieve panel-based prioritization
Input generation	Items often predefined based on literature or prior knowledge	Items generated in structured, often verbal group discussion	Open-ended crowdsourcing of facets from a diverse panel
Panel structure	Typically homogeneous experts	Small, face-to-face expert group	Context-driven, for instance experts, or diverse stakeholders (clinicians, other healthcare professionals, patients, etc…)
Process	Multiple rounds of questionnaires with controlled feedback	Idea generation, round-robin sharing, discussion, ranking	Facet elicitation, triage and structured recoding, refinement, consensus voting
Iterations	Multiple rounds with controlled feedback	Single meeting with discussion and ranking	Context-dependent Single or limited rounds; feedback includes recoding summaries and voting results
Interaction style	Anonymous, remote, no direct confrontation	In-person, structured interaction	Flexible (Anonymous, remote, no direct confrontation or hybrid with in-person, structured interaction)
Epistemic strengths	Efficient convergence; reduces dominance bias & enables global participation	Fast; ensures equal participation; captures discussion-based reasoning	Captures diverse conceptual facets; structures heterogeneous input; tailored for complex/rare disease contexts
Potential limitations	Time-consuming; limited generation of new items; risk of attrition	Limited scalability; potential group pressure; may miss less vocal perspectives	High interpretive component in recoding; panel-dependent; potential recall bias
Best-use case	Well-defined items requiring consensus	Small group prioritization or ranking	High-level, multidimensional concepts requiring structured elicitation, especially with sparse literature

In the ReCONNET-ELICIT approach, an expert panel is first tasked with generating a comprehensive list of all relevant facets associated with a high-level concept. Following this initial phase, the panel members individually vote to select which facets from the generated list should be included in the final high-level concept definition. This methodology emphasizes the importance of capturing a wide range of diverse perspectives through crowdsourcing of an expert panel, coupled with voting to refine the core elements.

In contrast, the nominal group technique (Table 1) typically involves a more interactive and face-to-face process, where experts first generate ideas independently and then share them with the group in a structured discussion [7]. After discussion, individual from the group ranks the ideas to establish consensus, thereby minimizing dominance bias and encouraging equal contribution among members. The nominal group method increases direct interaction and immediate deliberation within a dynamic environment.

The Delphi method, on the other hand, is characterized by its iterative, anonymous rounds of surveys distributed to experts across multiple stages [7]. In each round, experts provide their ratings, which are aggregated and shared with the group after each iteration. This process allows experts to reconsider their positions in light of the collective feedback, gradually moving towards consensus without the necessity of synchronous face-to-face interaction. The Delphi approach is particularly valuable when experts are geographically dispersed or when minimizing influence biases is crucial, as it relies on anonymity and controlled feedback to achieve convergence of opinions.

Therefore, while all three methods aim to facilitate expert consensus, the ReCONNET-ELICIT methodology merges the strengths of both traditional consensus-building approaches. It begins with a panel of diverse experts independently suggesting a wide range of facets related to a high-level concept, utilizing an online and asynchronous strategy. This approach is particularly advantageous when experts are geographically dispersed and operate across different schedules or timelines. After collecting and processing this initial data, ReCONNET-ELICIT allows the entire panel to participate in asynchronous voting to identify the most important facets for further development.

### In conclusion

the ReCONNET-ELICIT methodology distinguishes itself from conventional consensus-building approaches. By initiating the process with crowdsourcing from a diverse panel of experts, it enhances the efficiency, contextual applicability of facet elicitation. Furthermore, the collective voting process to determine the relevance of facets ensures that only pertinent aspects are prioritized by the group. This innovative approach is particularly well-suited to the context of rare connective tissue diseases (CTDs), which often involve limited resources and scarce data. In such settings, ReCONNET-ELICIT offers a structured framework, ensuring that elicited facets are both relevant and contextually appropriate from an expert perspective. Overall, this methodology facilitates the collective understanding of complex concepts, ultimately contributing to improved healthcare quality and accessibility for patients with rare diseases. The outputs reflect a consensus among a specific panel of experts at a given point in time and under particular clinical and methodological conditions and overall disease paradigm. They are therefore not to be interpreted as final, timeless, or universally valid definitions, but rather as structured, reproducible formulations intended to support specific purposes, such as research, clinical reasoning, or guideline development, within the given context, and may need updating as clinical knowledge evolves, new treatments emerge, or patient priorities shift. Future applications of ReCONNET-ELICIT may include tailoring care pathways, informing health policy, or even integrating artificial intelligence tools for facet extraction and prioritization.

## Data Availability

All data supporting the findings of this study are available within the paper.

## Declarations

### Disclaimer

The content of this publication represents the views of the authors only and it is their sole responsibility; it cannot be considered to reflect the views of the European Commission and/or the Health and Digital Executive Agency (HaDEA) or any other body of the European Union. The European Commission and the Agency do not accept any responsibility for use that may be made of the information it contains.
